# Performance enhancement in wavefront shaping of multiply scattered light: a review

**DOI:** 10.1117/1.JBO.29.S1.S11512

**Published:** 2023-12-20

**Authors:** Huanhao Li, Zhipeng Yu, Tianting Zhong, Puxiang Lai

**Affiliations:** a Hong Kong Polytechnic University, Department of Biomedical Engineering, Hong Kong, China; b Hong Kong Polytechnic University, Shenzhen Research Institute, Shenzhen, China; c Hong Kong Polytechnic University, Photonics Research Institute, Hong Kong, China

**Keywords:** wavefront shaping, optical phase conjugation, transmission matrix, optical speckles, optical modulation

## Abstract

**Significance:**

In nonballistic regime, optical scattering impedes high-resolution imaging through/inside complex media, such as milky liquid, fog, multimode fiber, and biological tissues, where confocal and multiphoton modalities fail. The significant tissue inhomogeneity-induced distortions need to be overcome and a technique referred as optical wavefront shaping (WFS), first proposed in 2007, has been becoming a promising solution, allowing for flexible and powerful light control. Understanding the principle and development of WFS may inspire exciting innovations for effective optical manipulation, imaging, stimulation, and therapy at depths in tissue or tissue-like complex media.

**Aim:**

We aim to provide insights about what limits the WFS towards biomedical applications, and how recent efforts advance the performance of WFS among different trade-offs.

**Approach:**

By differentiating the two implementation directions in the field, i.e., precompensation WFS and optical phase conjugation (OPC), improvement strategies are summarized and discussed.

**Results:**

For biomedical applications, improving the speed of WFS is most essential in both directions, and a system-compatible wavefront modulator driven by fast apparatus is desired. In addition to that, algorithm efficiency and adaptability to perturbations/noise is of concern in precompensation WFS, while for OPC significant improvements rely heavily on integrating physical mechanisms and delicate system design for faster response and higher energy gain.

**Conclusions:**

Substantial improvements in WFS implementations, from the aspects of physics, engineering, and computing, have inspired many novel and exciting optical applications that used to be optically inaccessible. It is envisioned that continuous efforts in the field can further advance WFS towards biomedical applications and guide our vision into deep biological tissues.

## 1 Introduction

Optical imaging in clear media, like free space, can be easily realized even with a single lens, with which light from an object can be easily relayed or conjugated to a detection plane with a one-to-one mapping,^1^ and a sharp image is therefore formed. It works well as long as ballistic photons dominate. Even with limited scattering events in thin biological tissues, i.e., photons propagate within one transport mean free path (TMFP), the portion of ballistic and/or quasiballistic photons are sufficiently large to be traced, which can be selected for high-resolution imaging via gating approaches, such as time gating-based optical computed tomography^2^ and spatial gating-based multiphoton microscopy.^3^ Aberrations from scattered photons (or nonballistic ones) induced by the tissue sample can be technically corrected via techniques, such as adaptive optics.^4^ However, if the optical path is way beyond the TMFP, such as in thick complex media or biological tissues, multiple scattering occurs and dominates the photon behavior due to the inherent spatial inhomogeneity of refractive index in the medium.^5^ In this scenario, the number of ballistic and quasiballistic photons exponentially decays rapidly as a function of sample thickness, as described by Lambert–Beer law, and eventually becomes negligible. Hence, the straightforward one-to-one mapping law that fits in clear media is broken. Indeed, the complex medium physically acts as a modulator that passively “shapes” the wavefront out of order, causing significant distortions. The conjugation between the object and image therefore becomes complicated: light travelling along different optical paths interferes with random phase shifts and as a result, light from one point on the object spreads widely on the detection plane. The object information is mapped to a “delocalized” speckle pattern, a visually random-distributed granny pattern, rather than a “localized” image of the target object. In this regard, the image quality is substantially degraded, and, in most cases, the seemingly random speckle pattern is the only representative that is optically accessible for analysis and processing.

**Fig. 1:**
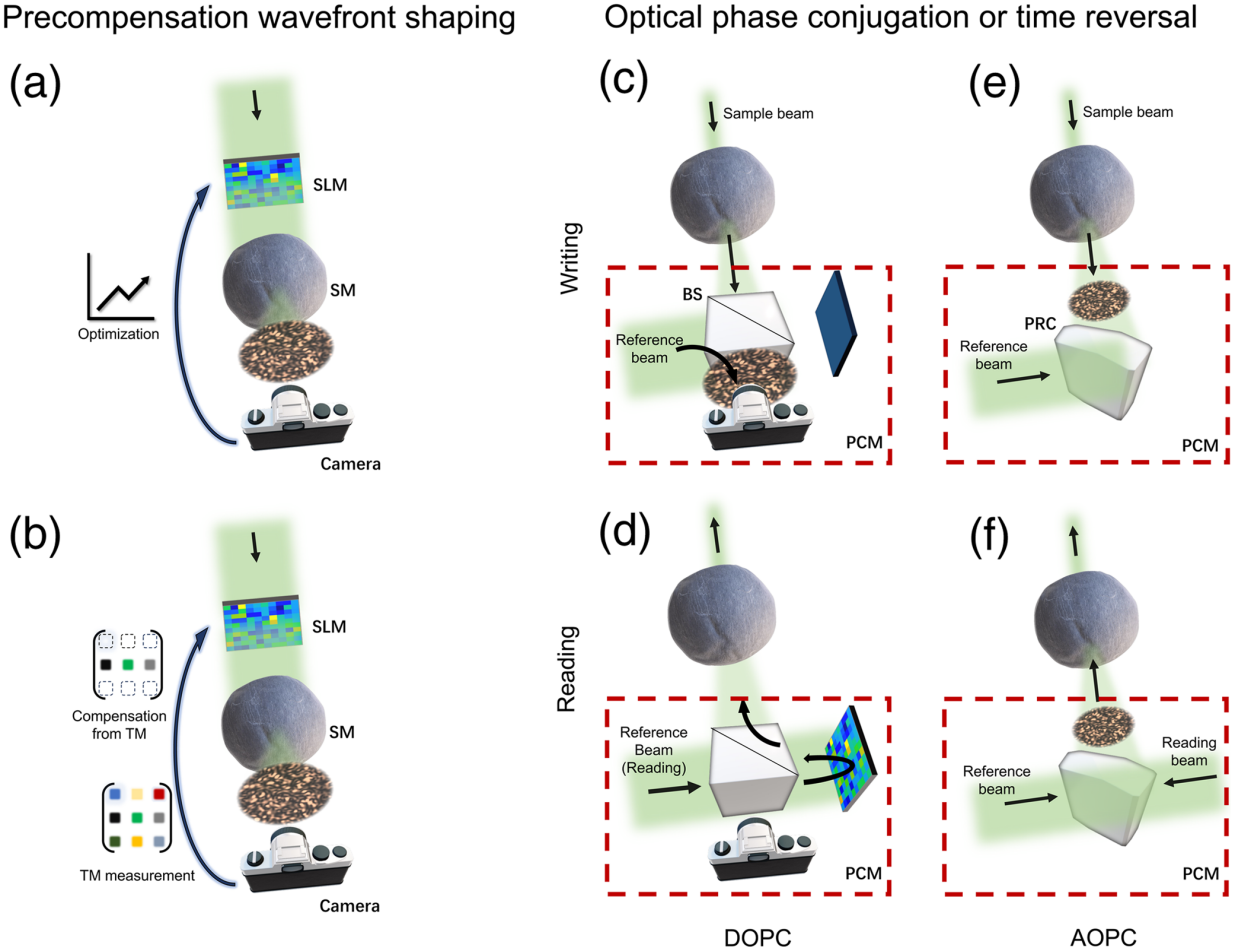
Illustration of the two types of WFS implementations. Precompensation WFS: (a) online iterative optimization and (b) TM-based method. (c)–(f) OPC or optical time reversal: (c), (d) DOPC; (e), (f) AOPC; (c), (e) hologram writing; and (d), (f) hologram reading. Abbreviations: SLM, spatial light modulator; SM, scattering medium; TM, transmission matrix; PCM, phase conjugation mirror; PRC, photorefractive crystal; OPC, optical phase conjugation; AOPC and DOPC: analogue OPC and digital OPC.

To overcome such chaos, wavefront shaping (WFS) has been proposed^6^^–^^10^ as a versatile tool to effectively turn the multiple scattering into controllable benefits for optical manipulation,^11^ imaging,^12^^–^^15^ and stimulation.^16^ By shaping the wavefront in order, usually with a spatial light modulator (SLM), the multiply scattered photons can be redirected and guided to arbitrary spatiotemporal coordinate of interest such as optical focusing.^17^^–^^19^ Depending on how wavefront is modulated, existing configurations of WFS can be categorized into two types, precompensation WFS and optical phase conjugation (OPC). For the former one [Figs. 1(a)–1(b)], the desired wavefront from the complex medium (e.g., to form a focus) is obtained by adjusting the optical wavefront input to the complex medium with an SLM: the modulation pattern (either phase and/or amplitude) is iteratively optimized based on the feedback from a physical or virtual detector, as shown in Fig. 1(a) or determined by the medium’s transmission matrix (TM), which is measured by probing the medium with a number of modulation patterns like Hadamard basis^15^^,^^20^^,^^21^ and random patterns,^22^^–^^25^ as shown in Fig. 1(b). For OPC [Figs. 1(c)–1(f)], also referred as optical time reversal, a phase conjugation mirror (PCM) in the system physically time-reverses the output wavefront from the complex medium back to the medium via two steps: (1) hologram writing [Figs. 1(c) and 1(e)]: the PCM records the hologram interfered between the output wavefront and a reference beam and then the sample beam carrying the output wavefront is off; (2) hologram reading [Figs. 1(d) and 1(f)]: a reading beam conjugated to the reference beam in Step 1 illuminates the PCM, generating a new wavefront conjugated to the original output wavefront, which traces back to the medium and eventually to the origin of the optical incidence. Notably, OPC can be realized in an either digital or analogue manner (called DOPC or AOPC accordingly), whose difference is the nature of the PCM. The PCM in DOPC is typically configurated with an SLM and a camera [Figs. 1(c) and 1(d)], both of which are strictly conjugated with a beamspliter, while the PCM is usually based on a photorefractive material, such as a photorefractive crystal (PRC), in AOPC [Figs. 1(e) and 1(f)].

Both types of implementations have been demonstrated to be capable of overcoming multiple scattering and achieving high-resolution optical focusing and imaging.^26^^–^^30^ Beyond that, each finds its own unique applications due to the subtle variations in these two realizations. For precompensation WFS, by properly defining the target to be optimized, it provides more flexible applications, such as polarization controller,^31^ intensity statistics refiner,^32^ dynamic holography,^15^ and nonlinear coupling.^33^^,^^34^ Regardless of the form of applications, exceptional performance requires numerous iterations for measurement and/or optimization on the order of $10^{3}$ to $10^{6}$,^21^^,^^26^ which is time-consuming and less compatible with *in vivo* applications if no sophisticated engineering and/or robust TM measurement are equipped. That is attributed to the fact that the whole shaping process must be accomplished within the optical correlation window (∼1  ms or less in living systems^35^); otherwise, the knowledge of medium status could be out of date and the compensation fails. In comparison, an OPC-based system can easily catch up with such a speed requirement as the time-reversed focusing can be done with just a single exposure, equivalently one iteration for its precompensation counterpart.^36^ Hence, exceptional demonstrations have been reported to focus scattered light through/inside dynamic medium,^37^^,^^38^ living tissues,^39^ and living animals.^27^^,^^40^

Nevertheless, further enhancement of WFS toward ideal refocusing of multiply scattered light is limited by the trade-off among three factors: degree of freedom (DoF), speed, and energy gain,^41^ which are mutually coupled by the shaping configuration, modulator (hardware), and algorithm (software). To envision further direction, this review will brief the progress and efforts to minimize such dilemma for both precompensation WFS and OPC and discuss how high-performance WFS benefits the fields of holography and biomedical imaging.

## 2 Modulators for Shaping

An SLM is the center of WFS. It provides a number (e.g., N) of independent modulation units for changing the phase or amplitude of eave wavelets, which determines the modulation efficiency and speed of the whole shaping process. Notably, the modulation DoF is usually set to be smaller than the total number of pixels on the SLM, since neighboring pixels can be grouped together as a modulation unit (or superpixel) for higher modulation efficiency. With a larger N, a finer wavefront can be shaped for better performance. For optical focusing, the performance is usually quantified by the intensity enhancement at the focus, characterized by the peak-to-background ratio (PBR, η). An ideal PBR can be estimated through $\eta_{\text{theory}}=\alpha (N-1)+1\overset{N\gg 1}{\to }\alpha N$, where α (i.e., modulation factor) is 1, $\frac{\pi }{4}$, $\frac{1}{\;\pi }$, and $\frac{1}{2\pi }$ for complex, phase-only, binary-phase, and binary-amplitude modulation, respectively.^42^ A larger N is often desired for better focusing performance, but it consumes a longer time for modulation optimization. Therefore, in practice, the selection of N is determined based on the balance between performance and time consumption.

The most commonly used modulator in WFS nowadays is liquid-crystal-on-silicon (LCoS) SLM for phase-only modulation with DoFs up to 1920×1080,^6^^,^^20^^,^^26^^,^^43^ due to its large modulation factor and handy engineering in the WFS system. For example, shaping the wavefront based on linear and nonlinear photoacoustic feedback, the optical intensity at the focus can be enhanced by ∼6000 times using an LCoS-SLM with N=20,736, while the whole wavefront optimization process took multiple hours.^26^ This speed is partially due to the slow refresh rate (60 Hz) of the LCoS SLM, which, in combination with a large N, sees many limitations in practice for precompensation WFS, as the WFS performance degrades significantly when the shaping period is beyond the correlation window.^35^ Fast SLMs, as listed in Table 1, are hence desired. It shall be clarified that in an experiment, the nominal maximum refresh rate of the modulator might not be reached due to different configurations and compatibility with the optical detectors. For example, the fastest modulator is grating light valves (GLV) with a refresh rate of 350 kHz, with which a TM-based focusing can be completed within 2.4 ms with 1088 DoFs and a camera as detector;^47^ employing an acousto-optic deflector (AOD) with 1260 DoFs as the SLM can achieve optical focusing within 10  μs with a 1-GHz APD detector;^48^ MEMS-based modulators, such as the MEMS-based phase-only SLM and the digital-micromirror devices (DMD), can function with a high speed driven by a field programmable gate array (FPGA) configuration. Recently, more and more implementations^14^^,^^21^^,^^29^^,^^49^^–^^52^ have selected DMD as the SLM in experiments, as it achieves a promising balance between the number of DoFs (i.e., performance) and refresh rate (i.e., consumption time), and its FPGA-based controlling unit is becoming more and more cost effective and user friendly. Notably, it should be clarified that DMD only provides binary amplitude modulation, but with Lee hologram intervention, full phase modulation can be achieved.^53^

**Table 1:** State-of-the-art of fast SLM used in WFS.

Modulator	Refresh rate	Reported DoFs	Modulation type	Modulation factor	Representative reference
NLC-SLM^a^	120 Hz	512 × 512	Binary-phase	1/π	36 and 44
MEMS^b^-based SLM	4.1 kHz	1024	Phase-only	π/4	45 and 46
MEMS-based DMD	23 to 30 kHz	1080 × 1920	Binary-amplitude	1/2π	14, 21, and 29
GLV^c^	350 kHz	1088	Phase-only	π/4	47
AOD^d^	∼80 kHz ^e^	1260	Phase-only	π/4	48

a NLC, nematic liquid crystal.

b MEMS, microelectromechanical system.

c GLV, grating light valves.

d AOD, acousto-optic deflector.

e This is an approximate number based on Ref. 48.

**Fig. 2:**
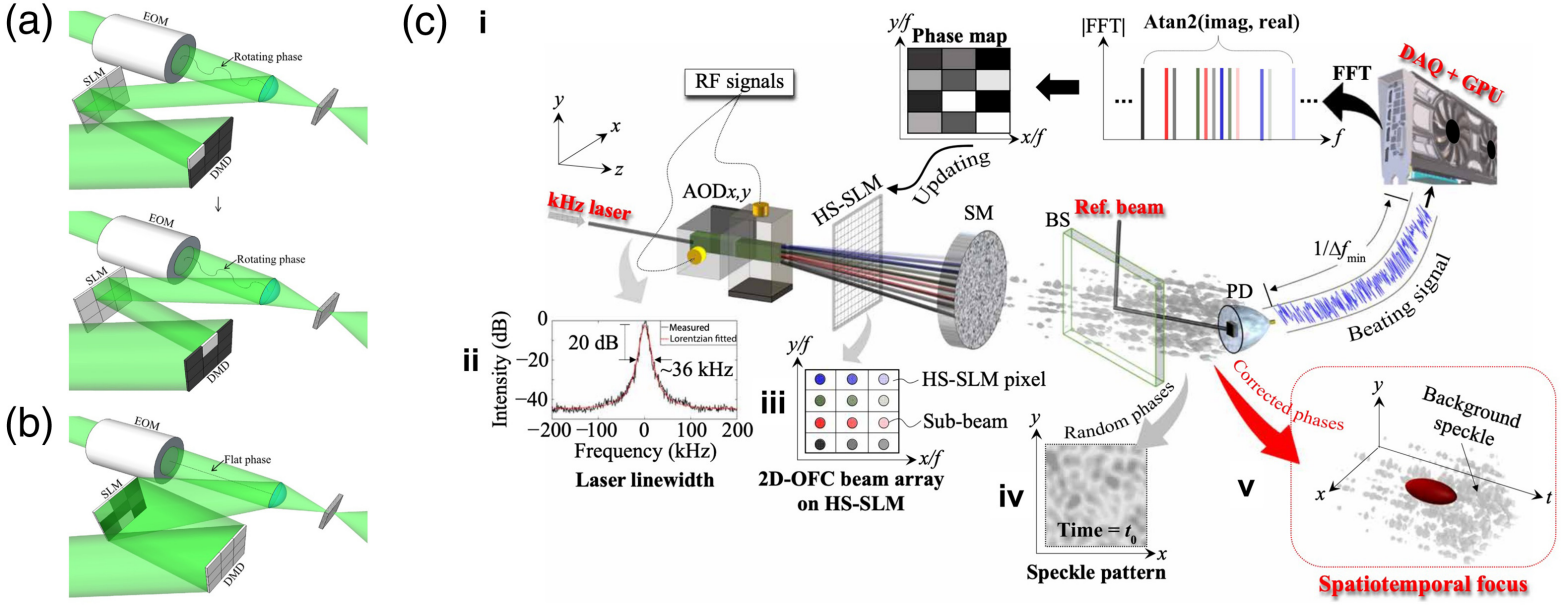
Representative realizations of employing more than one SLM for enhanced performance. Combination between a phase-only SLM and a DMD: (a) the phase measurement process, in which a flat phase is displayed on the SLM, and light reflected from each DMD superpixel illuminates a corresponding SLM superpixel while the EOM provides the phase modulation; and then (b) all DMD pixels are set to be on and optimized phase is displayed on the SLM, while the EOM remains a flat phase. (c) Hybrid modulation with an optical frequency comb, AOD, and an NLC-SLM. The HS-SLM (high-speed SLM) in (c) is a NLC-SLM. Images (a) and (b) are reproduced with permission from Ref. 54 and image (c) is reproduced with permission from Ref. 44.

To further enhance the performance, hybrid modulation has recently been proposed by combing different modulators via sophisticated pixel matching and control engineering. The first hybrid demonstration was based on the integration of an LCoS SLM and a DMD.^54^ As shown in Figs. 2(a) and 2(b), a DMD, assisted by an electro-optics modulator (EOM), was to measure the TM. An optimized phase pattern was calculated and then displayed on an LCoS SLM to achieve optical focusing with phase modulation but at the DMD speed. Later, the same group further developed another combination strategy^44^ as illustrated in Fig. 2(c): a beam array is compensated in a binary phase manner with a fast nematic liquid crystal (NLC) SLM, where subbeams are shifted with various frequencies and angles by a programmable two-dimensional optical frequency comb and two orthogonal AODs. With this setup, spatiotemporal focusing through a 1-mm-thick dynamic chicken breast and a living mouse was achieved in microseconds.

## 3 Precompensation WFS with High Performance

### 3.1 Fast and Accurate TM Measurement

**Fig. 3:**
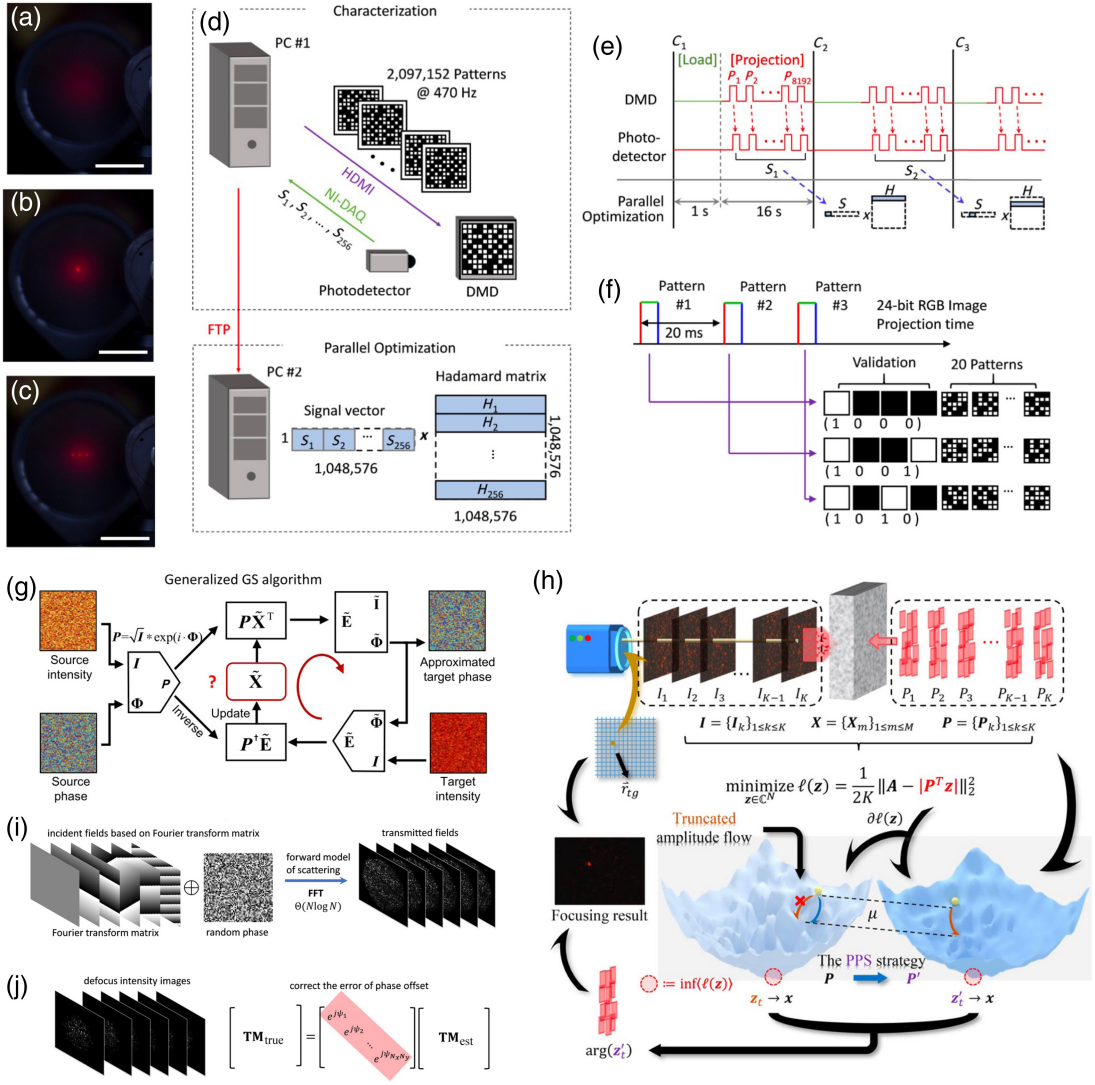
Representative TM methods with high performance. Demonstration of analytical real-part TM-based method:^21^ (a) speckles before optimization; (b) single focus after optimization; (c) three foci after optimization; (d) PC#1 is used to synchronize a DMD and a photodetector for pattern display and measurement and PC#2 is for the parallel optimization; (e) the data flow diagram; (f) DMD displaying workflow with a custom HDMI protocol, where the first four patterns are for error correction and the next 20 patterns are for probing patterns. (g) The flowchart of the GGS algorithm to retrieve the unknown TM.^25^ (h) A method of PPS-AF,^59^ using the PPS strategy to smooth the high-dimensional hyperplane (with many local minimums) to achieve fast convergence toward the global optimum. TM retrieval with fast Fourier transform (FFT): (i) encoding the Fourier transform matrix into the input wavefront allows FFT to accomplish the inverse algorithm of TM; and (j) phase error of TM can be corrected by the defocus images.^60^ Images (a)–(f), (g), (h), and (i), (j) are reproduced with permission from Refs. 21, 25, 59, and 60, respectively.

The TM-based schemes allow arbitrary focusing,^20^ raster scanning of the focus,^16^^,^^50^^,^^55^ image projection,^56^ and glare suppression^57^^,^^58^ on the detection plane without re-running the iterative optimization procedures.^20^ To measure a TM, analytical approaches commonly use orthogonal basis (e.g., Hadamard basis) with phase shifts to probe the medium, with required number of measurements ranging from 2N to 4N.^15^^,^^20^^,^^21^ Particularly, with DMD as the modulator, the number of measurements can be reduced to 2N−1 for binary modulation, and fast focusing based on a real-part TM can be achieved with PBR of more than 100,000 [Figs. 3(a)–3(c)] with 1,048,576 modes.^21^ It took ∼70  min with parallelized computation and fast data transfer via a custom HDMI protocol [Figs. 3(d)–3(f)]. One should be noted that TM measurement of 1 million modes based on an LCoS SLM (up to 60 Hz) with four phase-shift method could take at least $4\times \frac{10^{6}}{60}\;\mathrm{Hz}=4\times 16,666\;s=18.5\;h$. Statistical approaches for binary modulation were then developed with customized probing wavefronts and a complex TM can be obtained with convergence of optimization.^22^^–^^24^ However, due to the lack of customized phase profile to probe the medium in binary modulation, constraints applied to these optimizations are not strong enough to retrieve an accurate complex TM. Thus phase modulation is still preferred. Moreover, in practice, a DMD is often exploited as a phase modulator based on the Lee Hologram setting.^53^^,^^61^

It also should be noted that analytical solutions with orthogonal probing wavefront to obtain a complex TM based on phase modulation shows less adaptability to external noise during measurement, due to trivial physics assumptions.^15^^,^^20^ Iterative optimization with nonorthogonal probing wavefront is therefore preferred to converge the error and improve the measurement accuracy and adaptability. As shown in Fig. 3(g), by probing the medium with random wavefronts, a generalized Gerchberg–Saxton (GGS) algorithm 25 has been developed to iteratively optimize the unknown transfer function (i.e., the complex TM of an MMF), while conventional GS algorithm works with a known one, i.e., the Fourier transform between the target and source plane. It is robust to noise and perturbations, and due to the fast convergence of GGS, the computational time is orders of magnitude less than the previously proposed methods.^22^^,^^62^^,^^63^ Efforts are also paid to reduce the number of measurements to 2N with a method referred as probabilistic phase shaping guided amplitude flow (PPS-AF) as shown in Fig. 3(h).^59^ This method focuses on the statistics properties of the probing random wavefronts (i.e., uniform distribution for different phase ranges) instead of improving the algorithm itself, with which a complex TM of an MMF can be obtained with high accuracy. Moreover, as shown in Figs. 3(i) and 3(j), the probing random wavefront can be modified by a Fourier transform matrix to further improve the accuracy of TM.^60^

### 3.2 Fast and Adaptive Optimization Algorithms and Neural Networks

**Fig. 4:**
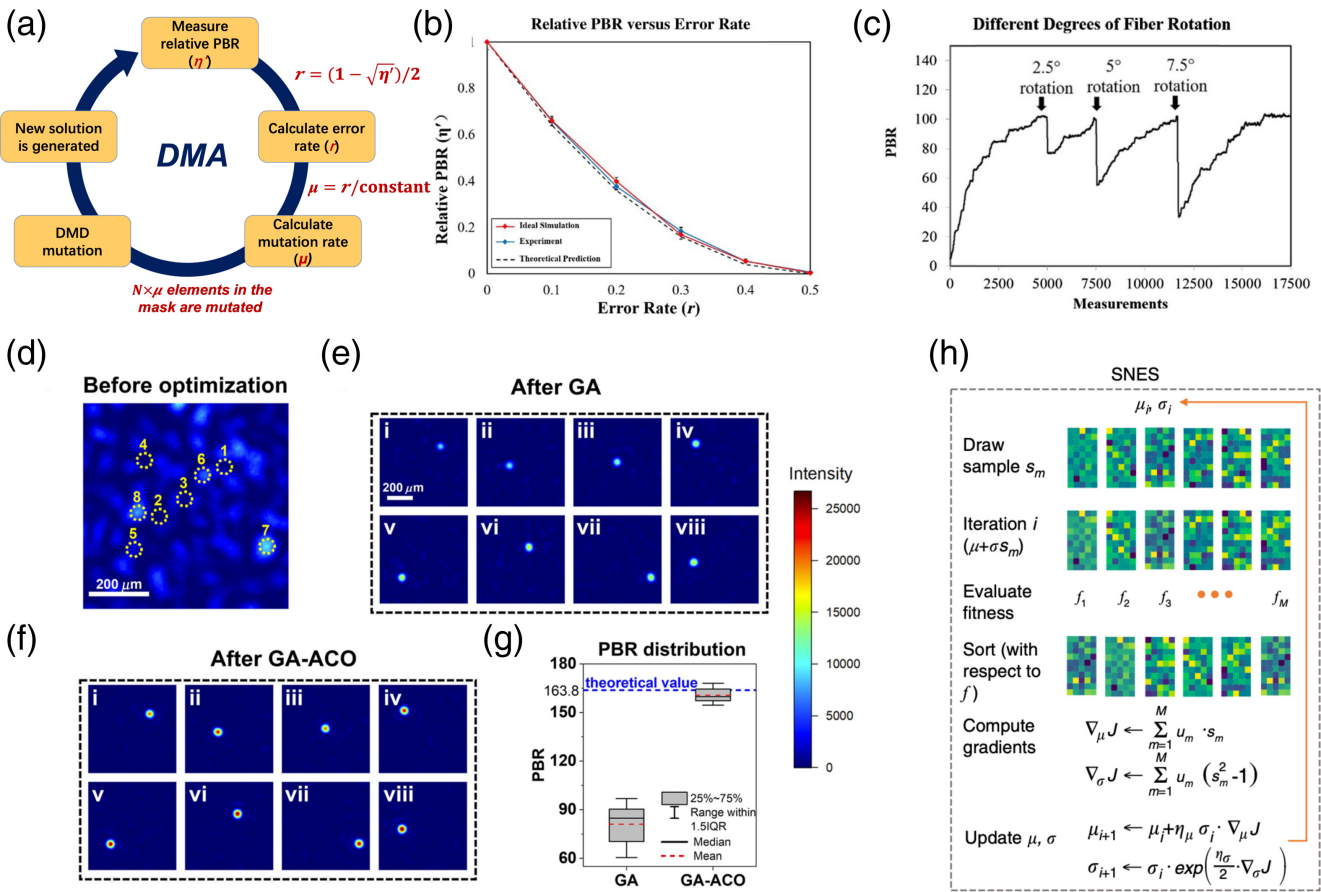
Representative adaptive and efficient precompensation algorithms. The dynamic mutation algorithm (DMA):^29^ (a) the flowchart of DMA; (b) the modulation square law validated by simulation and experiment; and (c) the recovery of PRB by DMA under multiple rotations applied to the MMF. GA-ACO algorithm:^30^ (d) speckles before optimization; (e), (f) focusing optimization with GA and GA-ACO, respectively; and (g) the statistics comparison for PBR between the GA and GA-ACO. (h) Flowchart of SNES algorithm.^71^ Images (a)–(c), (d)–(g), and (h) are reproduced with permission from Refs. 29, 30, and 68, respectively.

The application of TM methods, however, is inherently limited by the time span between the probing and compensation since the TM calculation occurs in between consumes time. If the computation power and engineering resources are limited, poor shaping performance is inevitable. Comparably, precompensation based on online iterative optimization algorithms can account for the distortions in a real-time manner. Early wavefront optimization algorithms were mostly migrated from the general optimization problems,^64^^,^^65^ among which genetic algorithm (GA)^66^ is one of the most popular choices due to its simple workflow yet general efficiency and compatibility with different optimization scenarios, such as focusing,^26^ imaging,^67^ and excitation of nonlinearity through multimode fibers (MMFs).^33^^,^^34^ However, when perturbations are applied to the medium or involved in the system, the efficiency of WFS reduces considerably. Thus many new developments in the field have focused on advancing the adaptability of algorithms. As a representative, a dynamic mutation algorithm^29^ has been proposed [Fig. 4(a)], whose optimization procedure is guided by an instantly obtainable binary-amplitude modulation error rate by monitoring the instantaneous PBR. The algorithm is based on a modulation square law [validated in Fig. 4(b)], which analytically bridges the modulation error rate (r) and the experimental real-time PBR ($\eta_{\mathrm{ex}}$) via $\eta^{\prime }=\eta_{\mathrm{ex}}/(N/2\pi )={\left(1-2r\right)}^{2}$ or $r=(1-\sqrt{\eta_{\mathrm{ex}}N/2\pi })/2$. That is, the number of binary superpixels that incorrectly modulates can be obtained in real time and be used to guide the optimization more efficiently. Such physics priori is independent on the medium status but is statistically general to the scenarios that elements in the TM of the medium follow the circular Gaussian distribution. It is shown in experiment, even when an MMF is rotated several times by different degrees, an optical focus can be well retained [Fig. 4(c)] without tuning the parameters in the algorithm or restarting the optimization procedure.

Such an error rate mechanism can also be included into other algorithms to combat the instability of complex media.^69^^,^^70^ Further, knowing the number of incorrectly modulating pixels is not enough as their coordinates are, too, important. Integrating ant colony optimization (ACO) into GA,^30^ a probabilistic map (to get either “ON” or “OFF”) for each binary-amplitude modulation pattern can be well estimated. With such information, tight and efficient focusing can be optimized [Fig. 4(f)] at different spatial coordinates, whose $\eta_{\mathrm{ex}}$ can be statistically optimized to the theoretical level [Fig. 4(g)], significantly surpassing the performance of pure GA [Fig. 4(e)] and other earlier algorithms. Specifically, for DMD as well, a separable natural evolution strategy (SNES) is proposed with continuous amplitude modulation realized by a multipixel encoding process [Fig. 4(h)]^68^ and, compared with peer DMD-based algorithms, the speed and PBR have been significantly improved and can be further used for pattern projection through an MMF.^71^

**Fig. 5:**
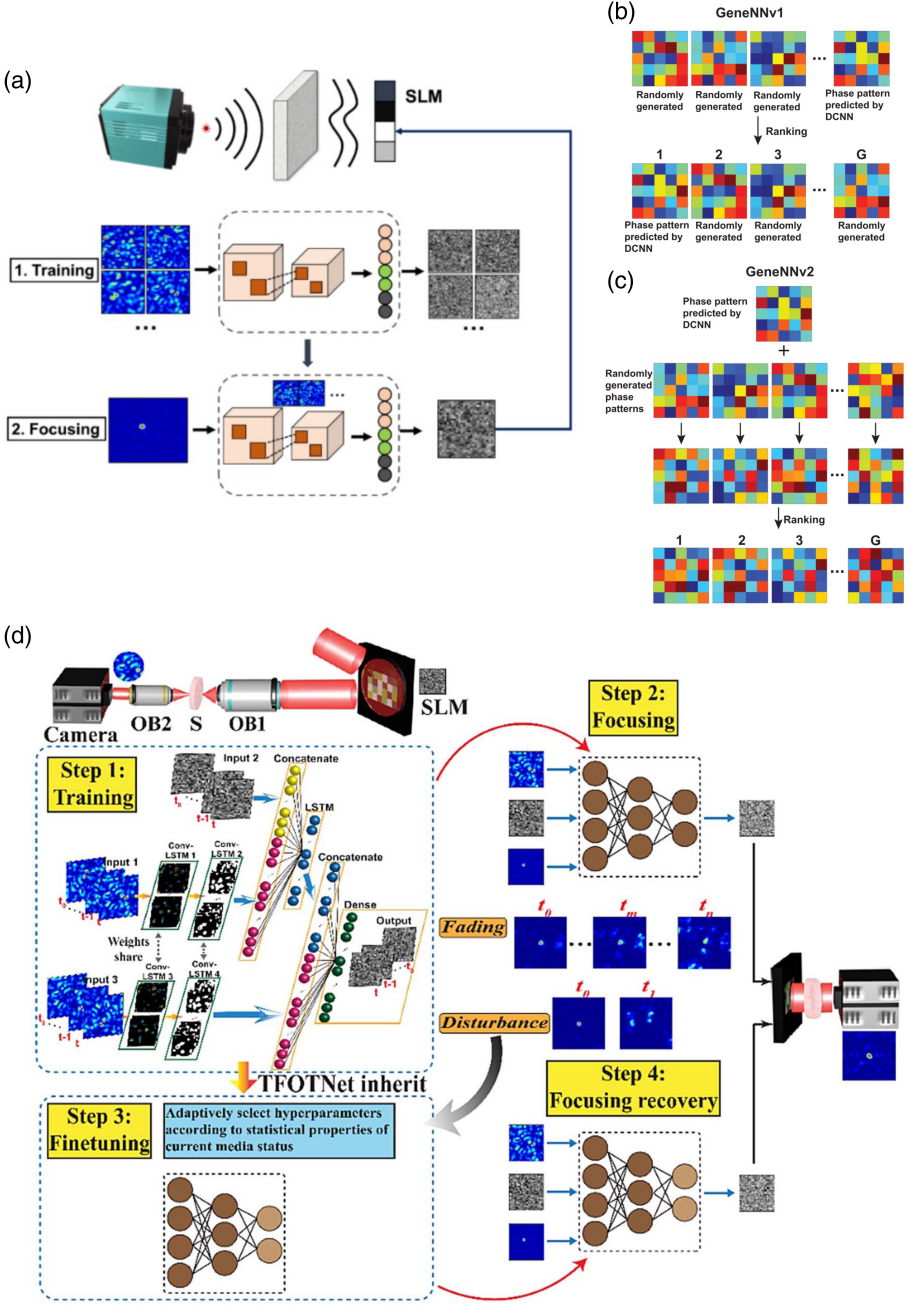
Representative learning-based precompensation WFS. A hybrid realization by reinforcing GA with DNN prediction:^74^ (a) the workflow, train the network first to output phase patterns, which will be combined with the phase patterns generated by GA; two different combinations (i.e., GeneNNv1 and GeneNNv2) between GA and DNN in (b) and (c). (d) Smart focusing through a moving diffuser with TFOTNet:^75^ the network is first trained for initial focusing and then fine-tuned by monitoring the time-varying focus/speckles for focusing recovery. Images (a)–(c) and (d) are reproduced with permission from Refs. 74 and 75, respectively.

Apart from conventional optimization algorithms, in the past few years, deep learning has also attracted intensive attentions in WFS, as a large amount of data are generated during wavefront optimization, especially when N is large (e.g., $>10^{3}$) and measurements/iterations with the same scale are needed. Deep neural networks (DNNs) can gain the knowledge of medium status, or equivalently the TM, by learning the training set, which has been demonstrated for complex media like a ground glass diffuser^72^ and an MMF^73^ for optical focusing and image projection. That said, the efficacy of the trained DNN, usually convolutional neural network, could decorrelate since the medium status keeps changing under unstable circumstances. To reinforce the adaptability of the DNN, optimization can be hybridized with a conventional optimization algorithm, such as GA as shown in [Fig. 5(a)]. In this trial, two DNNs including GeneNNv1 and GeneNNv2 [Fig. 5(b)] have been reported:^74^ the GA evolves the phase pattern output from the well-trained GeneNNs, which avoids random initialization, and the performance can be improved by 40% compared to a pure GA optimization. With a pure DNN, the same group also designed a Timely-Focusing-Optical-Transformation-Net (TFOTNet)^75^ to monitor and learn the changing intensity images of speckles/focusing over time and, more importantly, the TFOTNet is fine-tuned with data from several quasistatic periods (divided from the whole nonstationary period) to frequently refresh the memory units [Fig. 5(c)]. Such a scheme allows the TFOTNet gain real-time knowledge of the medium status, which further assures the maintenance of optical focusing as measured by the PBR through a moving scattering medium (ground glass). As seen, artificial intelligence (AI) can effectively enhance the performance of WFS and, hopefully, further development can be seen in the future to explore the application in biomedicine, which has been currently being reformed by the AI technologies.^76^

## 4 OPC with High Performance

Different from precompensation WFS, OPC does not need numerous iterations for optical focusing but usually a few or one exposure is sufficient to form a sharp and tight focus.^27^^,^^77^ Such a rapid response is essentially compatible with bio-applications whose correlation window is on the scale of milliseconds *in vivo*. The rapid response also allows for more DoFs being adopted for modulation without consuming too much time, and hence higher modulation efficiency could be expected. The discussion of recent progress in OPC will be unfolded from the perspectives of DOPC and AOPC, respectively.

### 4.1 DOPC with High Efficiency

**Fig. 6:**
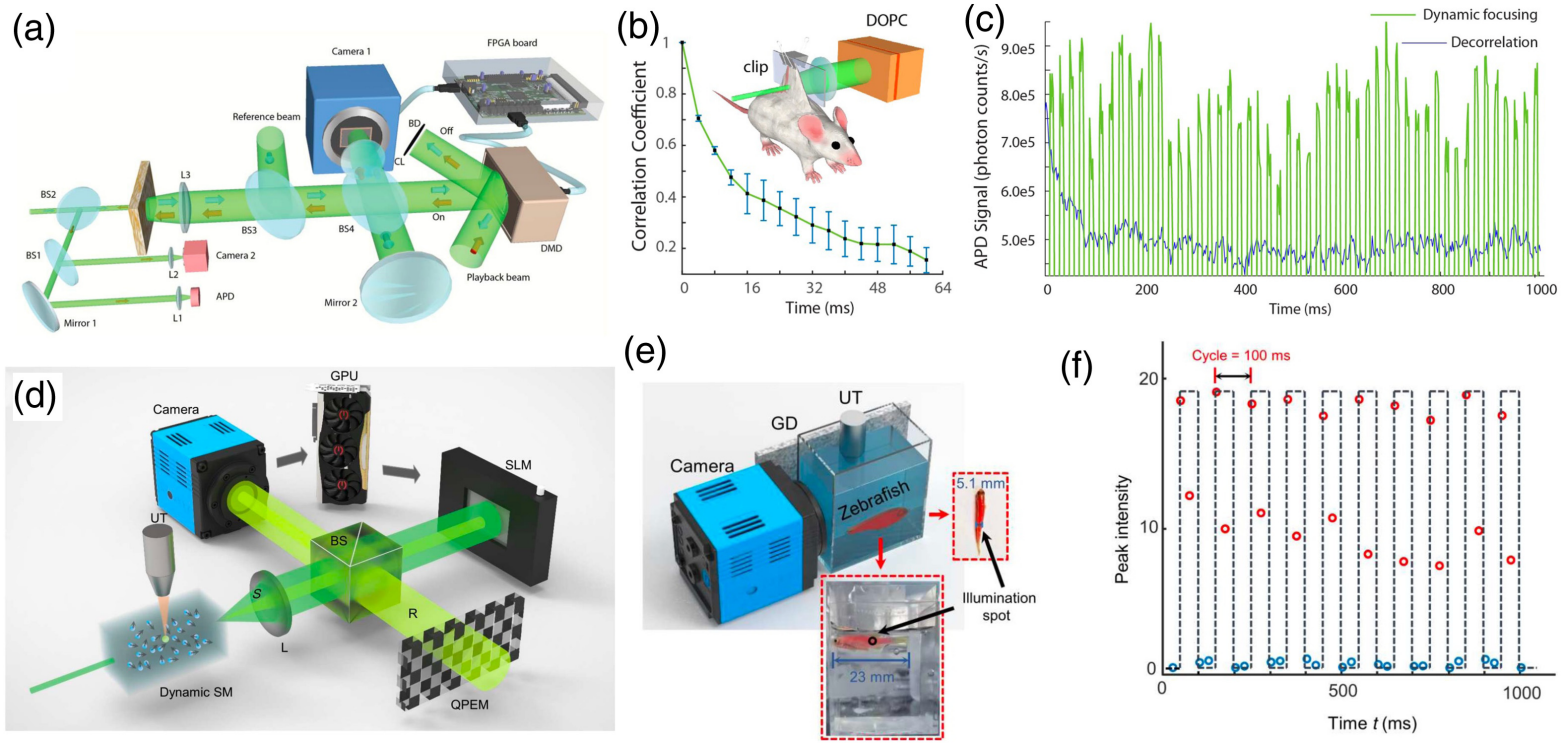
Representative fast DOPC dynamic focusing on millisecond scale. Dynamic focusing with a FPGA-DMD configuration in a DOPC system:^40^ (a) the schematic experimental setup; (b) decorrelation of the back on a living mouse; and (c) dynamic focusing during the decorrelation. High-speed single-shot TRUE focusing with full phase modulation:^77^ (d) DOPC setup with a QPEM and a GPU; (e) configuration for TRUE focusing in between the zebrafish and a ground glass diffuser; and (f) dynamic focusing realized in (e). Images (a)–(c) and (e), (f) are reproduced with permission from Refs. 40 and 77, respectively.

In principle, DOPC benefits from the employment of digital SLMs as the PCM, which sets no theoretical limit for the maximum reflectivity and selection of laser source (i.e., either continuous-wave or pulsed lasers is fine). In addition to such flexibility, DOPC solution therefore extracts wide visibility in imaging inside dynamic medium,^37^^,^^43^ edge enhancement,^78^ optogenetic modulation,^39^ as well as high NA focusing and imaging through a disordered metasurface.^79^ For example, toward *in vivo* optical focusing [Fig. 6(b)], with sophisticated engineering and alignment, a reported DOPC system with response time ∼5.3  ms with binary-amplitude modulation has achieved dynamic optical focusing through the back skin (2.3-mm thick) of a living mouse [Fig. 6(c)].^40^ A DMD is used as the SLM and its conjugated CMOS camera is controlled and triggered by an FPGA [Fig. 6(a)]. Similar with the data transfer used in Ref. 21, a customized HDMI protocol is used to encode 24 binary pixels into 24 bit RGB pixel. Yet with full phase modulation in DOPC, multiple shots are generally required to separate the ultrasonically tagged light due to the phase-shift method. To address that, a more recent study introduces a quaternary phase encoded mask (QPEM) to accomplish the four-step phase shifting in one shot [Fig. 6(d)],^77^ with a graphic processing unit (GPU) for data processing [Fig. 6(a)]. All this enables fast dynamic time-reversal ultrasonically encode (TRUE) focusing (<1  ms) between a 5.1-mm-thick zebrafish and a ground glass diffuser [Figs. 6(e) and 6(f)] with a single light exposure.

It is worthy of being noted that for optical focusing inside a complex medium, many interesting advances in DOPC have been achieved via ultrasonic modulation [e.g., focusing with TRUE light,^80^ time reversal of variance-encoded light,^81^ time-reversal ultrasound microbubble-encoded (TRUME) light^82^] and motion perturbation [e.g., focusing with time-reversed adapted-perturbations (TRAP)^43^ and controlled perturbations of magnetic particles^83^^,^^84^]. Wider applications of these implementations, however, have not yet been seen in the past few years due to the limited modulation efficiency of these guidestars.^85^

### 4.2 AOPC with High Efficiency

**Fig. 7:**
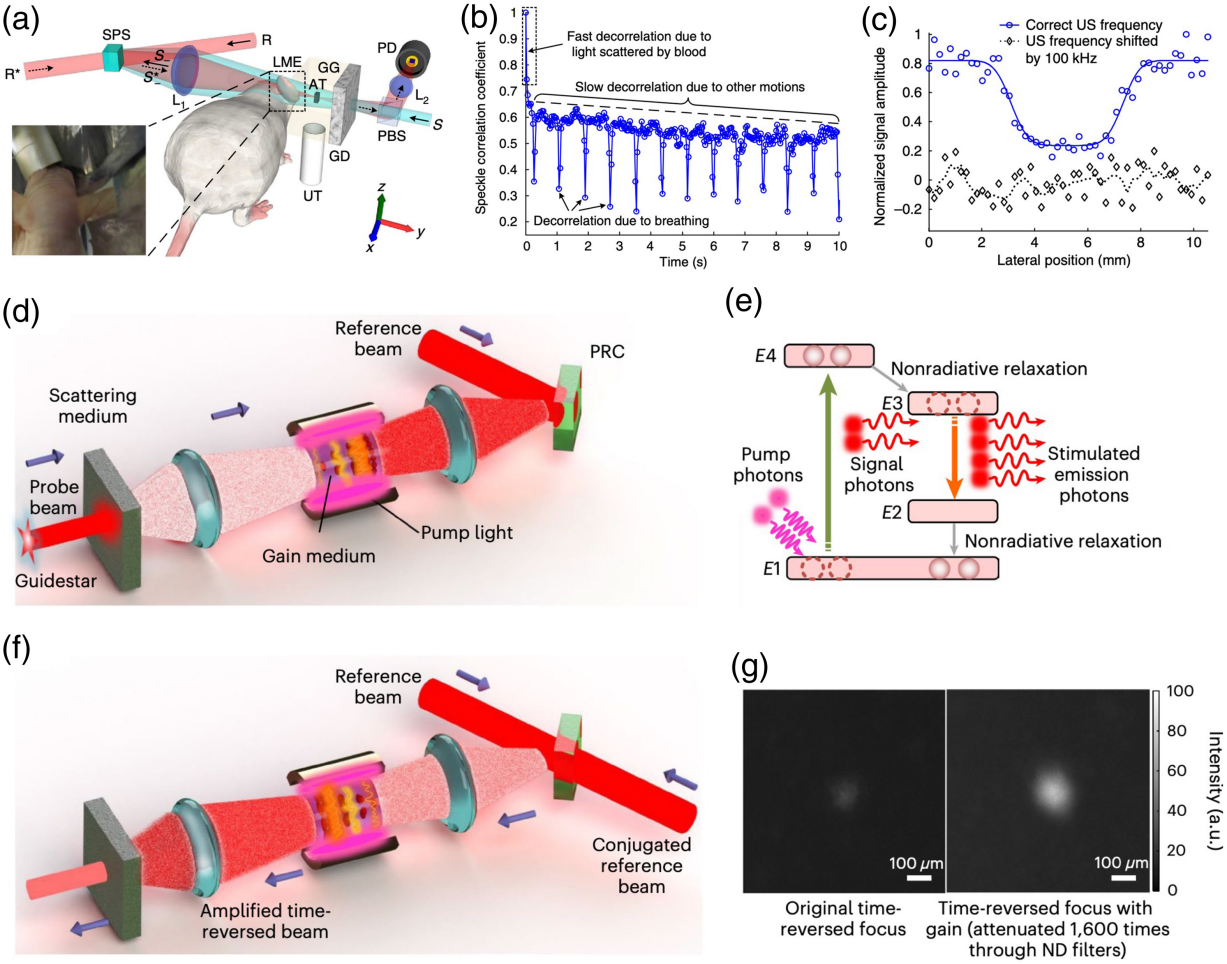
Representative realizations of efficient AOPC. Focusing inside dynamic scattering medium with near-infrared TRUE light in AOPC: (a) experimental schematic to focused diffused light and image an absorptive target between a diffuser and a living mouse; (b) decorrelation of speckles from a living mouse; and (c) one-dimension profile image of the absorptive target. High-speed and high-gain AOPC: (d) the recording process and (f) the reading process of the AOPC system; (e) diagram of the four-level gain medium of stimulated emission light amplification; and (g) focusing with and without the energy gain. Images (a)–(c), (d)–(g) are reproduced with permission from Refs. 27 and 87, respectively.

For AOPC, although recently it seems less popular than DOPC, employing PRC as the PCM in the system allows for a very large DoF and fast speed, which are weakly coupled. This is advantageous over DOPC where large DoF with a digital SLM inherently leads to extended duration for hologram recording and reading. For example, the DoF of PRC can be $10^{10}$ to $10^{11}$ in reported literature,^86^ while most SLMs to date have up to $2\times 10^{6}\;\mathrm{DoF}$ as shown in Table 1. In the meanwhile, the speed of AOPC is determined by the response time of the PRC, which can be improved by choosing appropriate materials and strengthening the illumination intensity, with limitation though. In practice, with a proper designed PRC, e.g., ${\mathrm{Sn}}_{2}P_{2}S_{6}$: Te 1% [denoted as SPS in Fig. 7(a), with $∼10^{7}$ DoFs] sensitive to wavelength of 790 nm being integrated in the AOPC system, TRUE focusing inside a dynamic complex medium with correlation time of 5.6 ms could be achieved.^27^ The setup could be further moved forward to image an absorptive target embedded in a tissue-mimicking phantom containing a living mouse ear [Fig. 7(b)] at ultrasonically determined resolution [Fig. 7(c)].

While promising, the application of AOPC is usually limited by the low-energy gain (e.g., $<10^{-3}$) due to low reflectivity of PRC ($∼10^{-3}$). In comparison, in DOPC, the reflectivity of SLM is on the order of $10^{-1}$ and an energy gain larger than unity can be achieved by increasing the power of the reading beam (below the damage threshold of the SLM) in the reading stage. A very recent study referred as high-gain and high-speed WFS (HGHS-WFS)^87^ addresses such low reflectivity of PRC through the introduction of stimulated emission light amplification [Fig. 7(e)] into an AOPC system. Encouragingly, optical focusing through a 4-mm thick tissue and a living mouse ear [Fig. 7(g)] is achieved with a unity gain ($10^{3}$ times of the gain obtained in previously reported AOPCs^27^^,^^86^^–^^88^) and a speed of ∼10  μs via a four-wave mixing scheme. Notably, as shown in Figs. 7(d)–7(f), the gain module is inserted between the scattering medium and the PRC (a gallium arsenide crystal). In operation, the low reflectivity of the PRC can be evaded and both the incident [in writing step, Fig. 7(d)] and outgoing [in reading step, Fig. 7(f)] components can be significantly enhanced. As a result, the net energy gain considering the ratio between the conjugated and original light beams can be scaled up to unity.

## 5 Discussion and Conclusion

As seen, dilemmas need to be tackled in precompensation WFS and OPC are quite different, which is determined by their mechanisms and, more directly, the number of exposures to obtain the expected modulation wavefront. The selection of modulator is more of concern in precompensation WFS since numerous iterations ($>10^{3}$) are inevitable, resulting in accumulated latency from all iterations. Therefore, urgent improvement of precompensation WFS comes to (1) acceleration: to speed up and accomplish the whole shaping process within the correlation window, typically on the scale of submilliseconds and milliseconds for living biological tissues and (2) adaptability: to strengthen the TM approach and iterative optimization to adapt to perturbations and noises when the whole process is proceeded in dynamic scenarios. For acceleration, regardless of the modulators, development of rapid-converging TM retrieval method is of focus from perspectives of probing wavefronts and retrieval algorithm, and sophisticated designs for both data transfer with custom protocol and workflow controlled by non-CPU units, e.g., GPU and FPGA, are mostly preferred. For adaptive enhancement, the optimization algorithms need to deal with the changing status of the medium and/or the system; optimization with physics priori reflecting the instant status, e.g., the binary modulation square law,^29^ can effectively update its own parameters with more precise guidance to optimal focusing. It also should be noted that monitoring the time-variant focusing enabled by a DNN with memory units can update the knowledge previously stored in the DNN, which may also enhance the capability towards dynamic focusing.^75^

In DOPC, due to the employment of digital SLMs, similar compromise shall be dealt with: more DoFs on the SLM slow down the optimization procedures, although the influence is a bit smaller. For example, in an identical DOPC system,^40^ $∼10^{6}$ DoFs leads to a latency ∼5.4  ms and $∼10^{5}$ DoFs to ∼1  ms, which is nonlinearly related. Nevertheless, the overall direction to improve the efficiency of OPC-based WFS focuses on accelerating the DOPC scheme, such as introducing QEPM to enable single-shot full phase modulation TRUE focusing within 1 ms,^77^ or enhancing the energy of phase conjugated light in AOPC by introducing a gain module in the system to accumulate the energy gain to approach or exceed the unity.^87^

Last but not the least, it must be admitted that till to date WFS still sees technical challenges for applications in thick and dynamic complex media, such as living biological tissues, due to insufficient understanding and solution of the multiply scattering process.^89^^,^^90^ Continuous efforts in merging, modifying, and advancing existing implementations toward more robust and more efficient performance could potentially inspire the second wave of innovations in WFS. Development of WFS in reflection mode could be a preference since transmission-mode imaging becomes less effective in deeper region of interest (ROI). Although only a “round-trip” reflection matrix for a scattering medium can be measured in common realizations, WFS integrated with fluorescence^91^ and time-gating^18^^,^^92^^–^^94^ approaches proves advantageous in discerning photons originating from the ROI, enabling precise positioning of an object plane and imaging targets within. Precalibrations for MMFs also benefit such reflection scenarios:^14^ premeasuring a set of MMF’s TMs and RMs under various bending states allows the tracking of the TM corresponding to the instantaneous bending state. This is achieved through analysis of the reflected signals, enabling point scanning at the MMF’s distal end during imaging. Notably, integration of MMF and WFS has seen further potentials. The employment of MMF provides a simplified model of complex medium with well-defined boundary conditions and has taken one-step further due to its negligible backscattering components and decomposable propagating modes.^12^ Consequentially, MMF, in combination with WFS, creates a unique minimally invasive (although not perfectly noninvasive) yet controllable optical pathway into deep biological tissue. That is why many of recent studies in WFS have adopted MMF as the complex medium and the light channel,^25^^,^^29^^,^^59^^,^^60^ with particular interests in MMF-based endomicroscopy.^14^^,^^50^^–^^52^

Collectively, this review has briefly included the recent technical advancements to enhance the performance of WFS, especially regarding specific dilemmas and developments for the two categories of WFS. Although there is still a long way to go before the perfection and wide applications of the technology, it is believed that the unique capability of manipulating multiply scattered light within or through thick complex media provides unprecedented opportunities for using light in a new horizon—noninvasive or minimally invasive high-resolution optical interactions and applications at depths in tissue, one of the biggest yet most promising challenges in biomedical optics.

## Data Availability

Data sharing is not applicable to this review article, as no new data were created or analyzed.
